# Metagenomic evidence of suppressed methanogenic pathways along soil profile after wetland conversion to cropland

**DOI:** 10.3389/fmicb.2022.930694

**Published:** 2022-09-20

**Authors:** Nannan Wang, Xinhao Zhu, Yunjiang Zuo, Jianzhao Liu, Fenghui Yuan, Ziyu Guo, Lihua Zhang, Ying Sun, Chao Gong, Changchun Song, Xiaofeng Xu

**Affiliations:** ^1^Key Laboratory of Wetland Ecology and Environment, Northeast Institute of Geography and Agroecology, Chinese Academy of Sciences, Changchun, China; ^2^University of Chinese Academy of Sciences, Beijing, China; ^3^Department of Soil, Water, and Climate, University of Minnesota, St. Paul, MN, United States; ^4^College of Life and Environmental Sciences, Minzu University of China, Beijing, China; ^5^Biology Department, San Diego State University, San Diego, CA, United States

**Keywords:** methanogenic pathways, methanogenesis, wetland cultivation, acetate pathway, CO_2_ pathway

## Abstract

Wetland conversion to cropland substantially suppresses methane (CH_4_) emissions due to the strong suppression of methanogenesis, which consists of various pathways. In this study, we evaluated the cultivation impacts on four predominant CH_4_ production pathways, including acetate, carbon dioxide (CO_2_), methylamines, and methanol, in a wetland and cultivated cropland in northeastern China. The results showed significant suppression of CH_4_ production potential and the abundance of genes for all four methanogenic pathways in cropland. The consistency between CH_4_ production and methanogenesis genes indicates the robustness of genomic genes in analyzing methanogenesis. The suppression effects varied across seasons and along soil profiles, most evident in spring and 0 to 30 cm layers. The acetate pathway accounted for 55% in wetland vs. 70% in the cropland of all functional genes for CH_4_ production; while the other three pathways were stronger in response to cultivation, which presented as stronger suppressions in both abundance of functional genes (declines are 52% of CO_2_ pathway, 68% of methanol pathway, and 62% of methylamines pathway, vs. 19% of acetate pathway) and their percentages in four pathways (from 20 to 15% for CO_2_, 15 to 9% for methylamines, and 10 to 6% for methanol pathway vs. 55 to 70% for acetate pathway). The structural equation models showed that substrate availability was most correlated with CH_4_ production potential in the wetland, while the positive correlations of acetate, CO_2_, and methylamine pathways with CH_4_ production potential were significant in the cropland. The quantitative responses of four CH_4_ production pathways to land conversion reported in this study provide benchmark information for validating the CH_4_ model in simulating CH_4_ cycling under land use and land cover change.

## Introduction

Wetlands are the major biological source of atmospheric methane (CH_4_), and the annual emission is estimated as high as 104–124 Tg C per year across the globe (Kirschke et al., 2013; Rosentreter et al., 2021). Human activities-induced natural wetlands loss has reduced approximately 54% of CH_4_ emissions from 1949 to 2009 across China (Xu and Tian, 2012), 86% of which was caused by land conversion (Li et al., 2012). Even though the massive suppression of CH_4_ emission is driven by methanogenesis depression, little genomic evidence has been reported (Masuda et al., 2018; Keuschnig et al., 2022). Moreover, methanogenesis processes involve different pathways (Conrad, 1996; Xu et al., 2015, 2016), and how each methanogenic pathway responds to cultivation remains elusive.

There are mainly four methanogenic pathways: acetate, carbon dioxide (CO_2_)/hydrogen (H_2_), methanol, and methylamines pathways (Deppenmeier, 2002; Liu and Whitman, 2008; Lyu et al., 2018). In wetland and rice fields, the acetate pathway is dominant, accounting for approximately two-thirds of the CH_4_ production (Conrad, 1999; Ferry and Kastead, 2007; Bogard et al., 2014). The CO_2_ pathway, driven by hydrogenotrophic methanogens, dominates in the environment with a high H_2_ concentration (Thauer et al., 2008; Lyu et al., 2018). Most studies emphasize the roles of acetate and CO_2_ pathways (Conrad, 2020), while less frequently considering methanol and methylamines pathways. Recently, the methanol and methylamines reduction pathways have been found in varied habitats such as marine and freshwater wetlands (Vanwonterghem et al., 2016; Narrowe et al., 2019), and also proved that methanol and methylamines are different in their contributions to CH_4_ emissions (Narrowe et al., 2019; De La Cuesta-Zuluaga et al., 2021). Overall, these four pathways are all significant in contributing to CH_4_ production. Moreover, their responses to cultivation might vary due to various physiological processes (Gonzalez-Gil et al., 2001; Demirel and Scherer, 2008). Therefore, it is vital to link the potential of CH_4_ production with the metabolic potential of four methanogenic pathways (Pedersen et al., 2018). Studying the presence of functional genes is a good approach to examining the microbial potential for CH_4_ production. It is also possible to distinguish functional genes in four pathways since most enzymes driving four pathways of CH_4_ production are different.

Organic matter quality and temperature are the most important controls on methanogenesis and its pathways (Holmes et al., 2014; Deng et al., 2019). On one hand, easy degradable organic carbon increased in cultivated cropland through soil organic carbon decreased compared with marshland (Song et al., 2012). It indicated that wetland cultivation would enhance the labile carbon fraction. Theoretically, ~67% of the CH_4_ production has been attributed to acetoclastic methanogenesis, while the other 33% comes from reducing CO_2_ when the cellulose is completely degraded (Conrad, 1999). Both microbial and isotopic evidence proved that the contribution of the acetate pathway would increase when the substrates are more labile such as fresh input of litter (Ji et al., 2018), while the CO_2_ pathway dominants in oligotrophic wetlands such as northern bogs and lake sediments, in which the organic matters are recalcitrant for degradation (Hines et al., 2001; Horn et al., 2003; Rooney-Varga et al., 2007). The quality of organic matter can also explain the relative contribution of the CO_2_ pathway increases with depth because deeper layers of organic matter are increasingly recalcitrant (Zepp Falz et al., 1999; Picone et al., 2020). On the other hand, the temperature would be more variable without flooding. Previous studies proved that even though both CO_2_ and acetate pathways decrease with temperature, the inhibiting effect of low temperature on hydrogenotrophic methanogenesis is more significant than that in acetoclastic methanogenesis (Schulz and Conrad, 1996; Chin et al., 1999; Chen et al., 2020). The relative contribution of the CO_2_ pathway has also been found to decrease with temperature both in sediment and rice field soils (Metje and Frenzel, 2005; Nozhevnikova et al., 2007; Zhang et al., 2015) due to the adaptation of acetogens and thermodynamics of syntrophic fatty acid oxidation, which result in relatively more production of acetate than H_2_ (Glissman et al., 2004; Conrad, 2020).

Thus, this study aims to (1) assess whether soybean cultivation affects functional genes regarding four methanogenic pathways and their relative contributions; and (2) understand the meteorological and edaphic effects on four pathways and their proportions across seasons and soil profiles. To achieve these, we quantify the relative abundance of functional genes involved in four methanogenic pathways based on metagenomes across four seasons and soil profiles. The controls of soil edaphic and micrometeorological characters were also determined. We expect that (1) all four pathways would be inhibited after cultivation but to different extents; (2) cultivation would enhance the contribution of the acetate pathway rather than the CO_2_ pathway because the substrate in cropland is more labile than that in the wetland; and (3) the seasonal variation of the CO_2_ pathway was more significant than the acetate pathway in cropland because of its temperature sensitivity.

## Materials and methods

### Site description and sampling

Soil cores were taken from the Sanjiang Mire Wetland Experimental Station located in Sanjiang Plain (133°31′E, 47°35′N) in Tongjiang City, northeastern China. Sanjiang Plain is the largest freshwater wetland in China, with ~10,560 km^2^ in area (Shi et al., 2020). This area is characterized by a temperate humid and subhumid continental monsoon climate. The mean annual temperature is 2.5°C, and the mean annual precipitation is 500–600 mm. A marshland and adjacent cropland were selected as our sampling sites, representing natural wetland and cultivated cropland. The wetland's surface is covered by 30–35 cm of water, and dominated by *Carex meyeriana, Carex lasiocarpa*, and *Deyeuxia angustifolia* communities. Cropland has been converted from the same wetland in 1996, and Soybean [*Glycine max* (L.) Merr.] was cultivated in cropland from May to October every year without any fertilization and stand litter removal (Zhu et al., 2021). The soil texture in our study site is silty loam.

Soil cores were collected from natural wetland and cultivated cropland in autumn (10–12 October 2019), winter (27–29 December 2019), spring (13–15 May 2020), and summer (19–21 July 2020), respectively. Three replications in each ecosystem, and we totally have 24 soil cores. Every soil core was of depth 100 cm and divided into 10 sections (0–10, 10–20, 20–30, 30–40, 40–50, 50–60, 60–70, 70–80, 80–90, and 90–100 cm). A total of 240 samples were packed in polyethylene bags immediately and brought back to the laboratory with ice packs within 24 h for further analysis.

### Measurement of CH_4_ production potential and CH_4_ flux data collection

Potential CH_4_ production rates were determined by lab incubation. About 20 g of dry weight soil and 100 ml ddH_2_O were put into a bottle. Then, the bottle was flushed with nitrogen gas for 5 min to keep the anaerobic condition. Gas was extracted immediately when the bottle was sealed using injectors with a three-way valve on the lid. Every soil sample has three replications. Then, all the bottles were incubated at 30°C for 5 h. After the incubations, gas was extracted again using injectors with three-way valves. The CH_4_ concentration was measured by chromatography (Agilent 7890, USA). The changes in CH_4_ concentration, with normalized to dry weight soil, represent CH_4_ production potential. Local CH_4_ flux includes measurement by the chamber in our experimental sites (Hao, 2005; Liu et al., 2011). The global CH_4_ flux data were collected from a global scale literature survey. Specifically, we searched data from journal articles through the “Web of Science” database until 5 December 2021, with no restriction on the publication year. The keywords are “(Wetland^*^ OR Swamp^*^ OR Marsh^*^) AND (Reclamation OR Culti^*^ OR Land-use-change^*^ OR conversion^*^ OR transition^*^) AND (methane^*^ OR CH${}_{4}^{*}$ OR methano^*^ OR mcrA OR pmoA).” A total of 876 papers matched the keywords. The criteria for selecting proper studies were as follows: (1) both pristine wetland and conversed farmland were included in the same study area within the same measurement time; and (2) the variables refer to CH_4_ flux. A total of 438 pairs of observed variables taken from 23 publications were regarded as global CH_4_ flux.

### Measurements of environmental factors

Soil edaphic characters, including soil total carbon (T.C.), total nitrogen (T.N.), total phosphorus (T.P.), and total sulfur (T.S.), were measured using high-temperature combustion and heating digestion methods, respectively (Zhu et al., 2021). Soil microbial biomass carbon (MBC), nitrogen (MBN), phosphorus (MBP), and sulfur (MBS) were measured using the fumigation method (Zhu et al., 2021). Soil water content (SWC) and soil temperature (S.T.) are involved in the microion methods' meteorological characteristics. Soil water content was determined by the drying method. Supporting soil temperature was obtained from the long-term automatic weather stations in wetland and cropland.

### Soil DNA extraction and sequencing

DNA was extracted according to the protocol using fastDNA^®^ Spin Kit (M.P. Biomedicals, Inc., CA, USA). We extracted 214 DNA samples, and 26 soil samples failed to extract DNA. Metagenomic libraries were constructed using NovaSeq Reagent Kits from all samples with ~400 bp insert sizes. Sequencing 150 bp reads were performed on Illumina NovaSeq 6000 (Illumina Inc., San Diego, CA, USA) platform at Majorbio Bio-Pharm Technology Co., Ltd. (Shanghai, China). Totally 1.6 billion paired-end reads were produced, resulting in an average of 50 million reads per sample.

### Bioinformation and statistics

After removing adaptor sequences and trimming, high-quality reads were obtained by removing low-quality reads, including reads with N bases, length < 50 bp, and quality < 20 using fastp (https://github.com/OpenGene/fastp, version 0.20.0) (Chen et al., 2018). Those high-quality reads were used to assemble contigs using MEGAHIT (https://github.com/voutcn/megahit, version 1.1.2) (Li et al., 2015). Contigs lengths over 300 bp were selected to identify open reading frames (ORFs) using MetaGene (http://metagene.cb.k.u-tokyo.ac.jp/) (Noguchi et al., 2006), and the predicted ORFs with lengths over 100 bp were translated into amino acid sequences using the NCBI translation table (http://www.ncbi.nlm.nih.gov/Taxonomy/taxonomyhome.html/index.cgi?chapter=tgencodes#SG1). The CD-HIT (http://www.bioinformatics.org/cd-hit/, version 4.6.1) was used to construct a non-redundant gene catalog. Then mapped high-quality reads to the non-redundant gene catalog with 95% identity using SOAPaligner (http://soap.genomics.org.cn/, version 2.21) (Li et al., 2008) and evaluated gene abundance in each sample. Representative sequences of the non-redundant gene catalog were annotated based on the NCBI NR database using blastp as implemented in DIAMOND version 0.9.19 with an e-value cutoff of 1e^−5^ using Diamond (https://www.bluenile.com/cn/en/diamond-search, version 0.8.35) for taxonomic annotations. The genes annotations were conducted using Diamond (Buchfink et al., 2015) against the Kyoto Encyclopedia of Genes and Genomes (KEGG) database (https://www.genome.jp/kegg/, version 94.2) with an e-value cutoff of 1e^−5^.

We calculated the proportion of genes in 1 million observations per sample (ppm). Four pathway modules of methanogenesis were identified: acetate pathway (M00357), H_2_/CO_2_ pathway (M00567) (shorted as CO_2_ pathway in the following text), methanol pathway (M00356), and methylamines pathway (M00563), according to KEGG database (Supplementary Table S1). The abundances of 12 ECs (EC number system based on enzyme nomenclature) encoding enzymes involved in methane production metabolism were also estimated (Supplementary Figure S1A). All data were log-transformed before comparison. Then, assuming that four pathways were 100%, the percentages of four pathway genes were calculated to identify each pathway's gene ratio. The correlations between the percentages of four pathways to micrometeorological and edaphic characteristics were estimated. Micrometeorology refers to soil temperature and soil water content. Edaphic characteristics here and after that included T.C., T.N., T.P., T.S., and C/N ratio. Principal component analysis was used to reduce dimensionality. Methanogens community structures were determined by non-metric multidimensional scaling (NMDS) based on the “Bray–Curtis” distance in order level. The abundances of eight mainly methanogenetic orders were also compared.

The CH_4_ fluxes were added 10 before log-transformation and calculation to avoid negative values. Differences between natural wetland and cultivated cropland were determined with the Kruskal–Wallis test. Dunn's test was used for multiple comparisons and the *P*-value adjustments method was “Bonferroni.” The linear regression analysis between percentages of four pathways and micrometeorological and edaphic characters was performed using a package of lme4 in R version 4.0.2.

Structural equation modeling (SEM) analysis was employed to identify the direct and indirect influence of micrometeorological and edaphic characteristics, biomass, and functional genes on CH_4_ production potential. Before SEM analysis, the bivariate relationships between all variables with simple linear regressions were checked to ensure the appropriateness of the linear models. The adequacy of the model was determined by the χ^2^ test, root mean square errors of approximation (RMSEA), and Goodness-of-fitness index (GFI). All data used in SEM were normalized. Biomass is referred MBC, MBN, MBP, MBS, and MBC/MBN ratio. The SEM was performed by a package of “lavaan” in R version 4.0.2.

## Results

### Methane flux and production potential in response to cultivation

**Figure 1:**
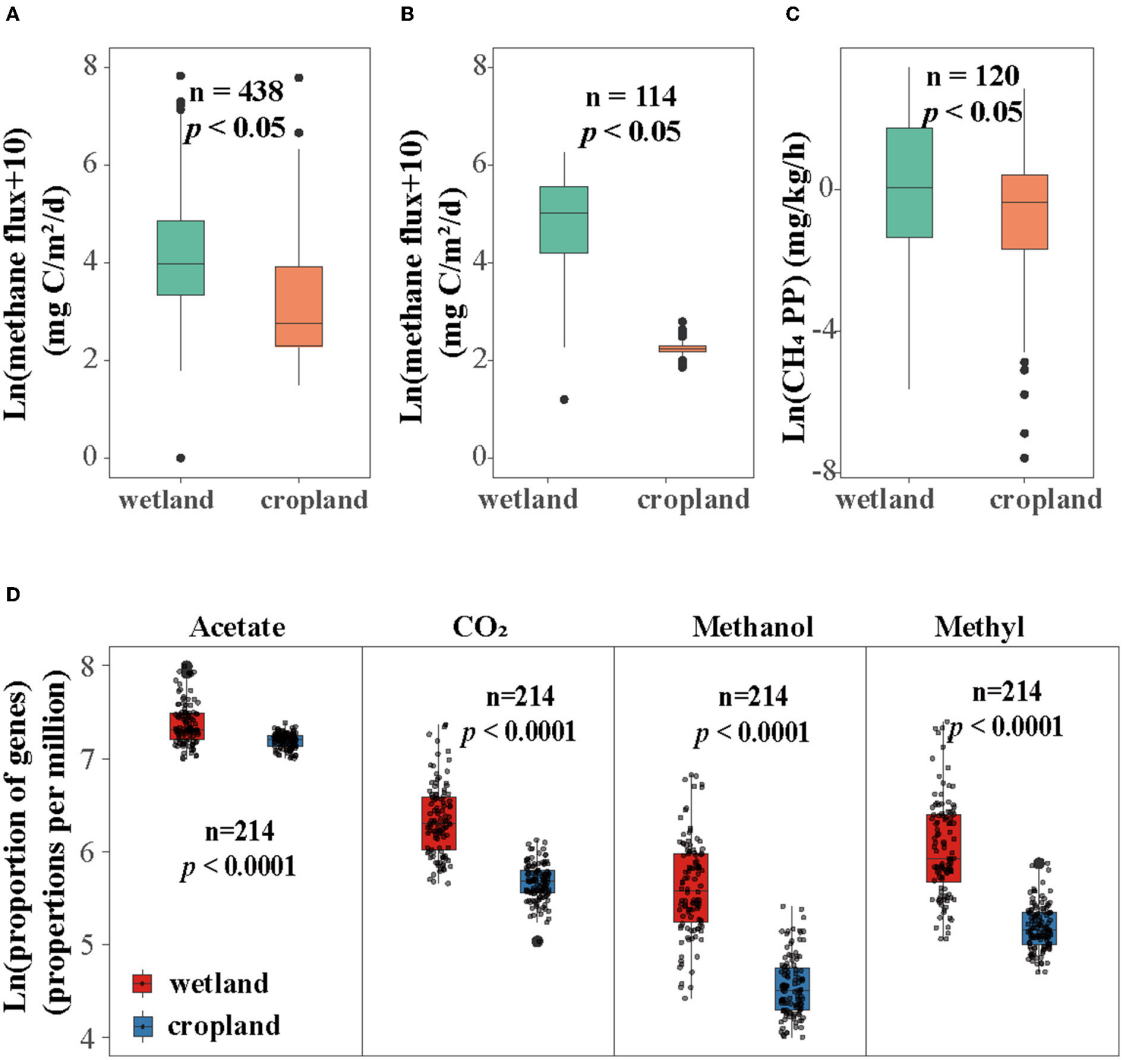
Influence of cultivation on methane flux in **(A)** global scale, **(B)** local scale, **(C)** methane production potential (CH_4_ PP), and proportion of genes in four methane production pathways. **(D)** Relative abundance of four pathways including acetate pathway, CO_2_ pathway, methanol pathway, and methylamines (short for methyl) pathway. The proportion of four pathways was the relative abundance of functional genes involved in four pathways calculated as parts per million (ppm) and then log-transformed.

CH_4_ flux on global and local scales and production potential were estimated. Our results showed that CH_4_ flux and production potential were suppressed in cultivated cropland (Figures 1A–C). Specifically, CH_4_ flux on the local scale (Sanjiang Plain) decreased after cultivation from CH_4_ emission (6.75 mg C m^2^ h^−1^) to slight uptake (-0.023 mg C m^2^ h^−1^) (Figure 1B). CH_4_ production potential also reduced significantly in cultivated cropland (Figure 1C). Moreover, the CH_4_ flux on the global scale decreased from 43.58 mg C m^2^ h^−1^ in the natural wetland to 5.86 mg C m^2^ h^−1^ in the cropland (Figure 1A).

### Cultivation impacts methanogenic pathways and their proportions

**Figure 2:**
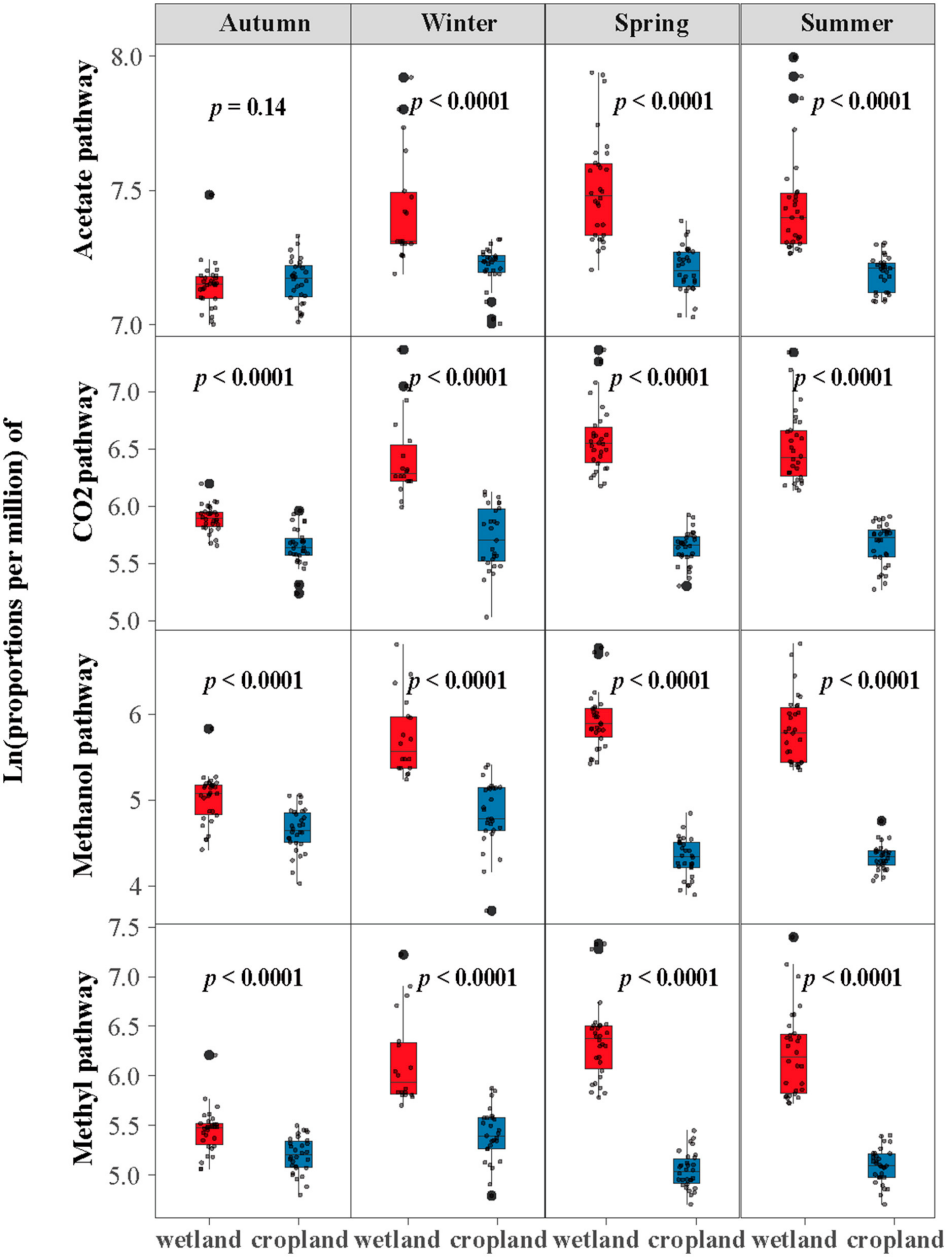
Cultivation impacts the proportions of genes in four methane production pathways across seasons. Log-transformation with base e was used before comparison.

**Figure 3:**
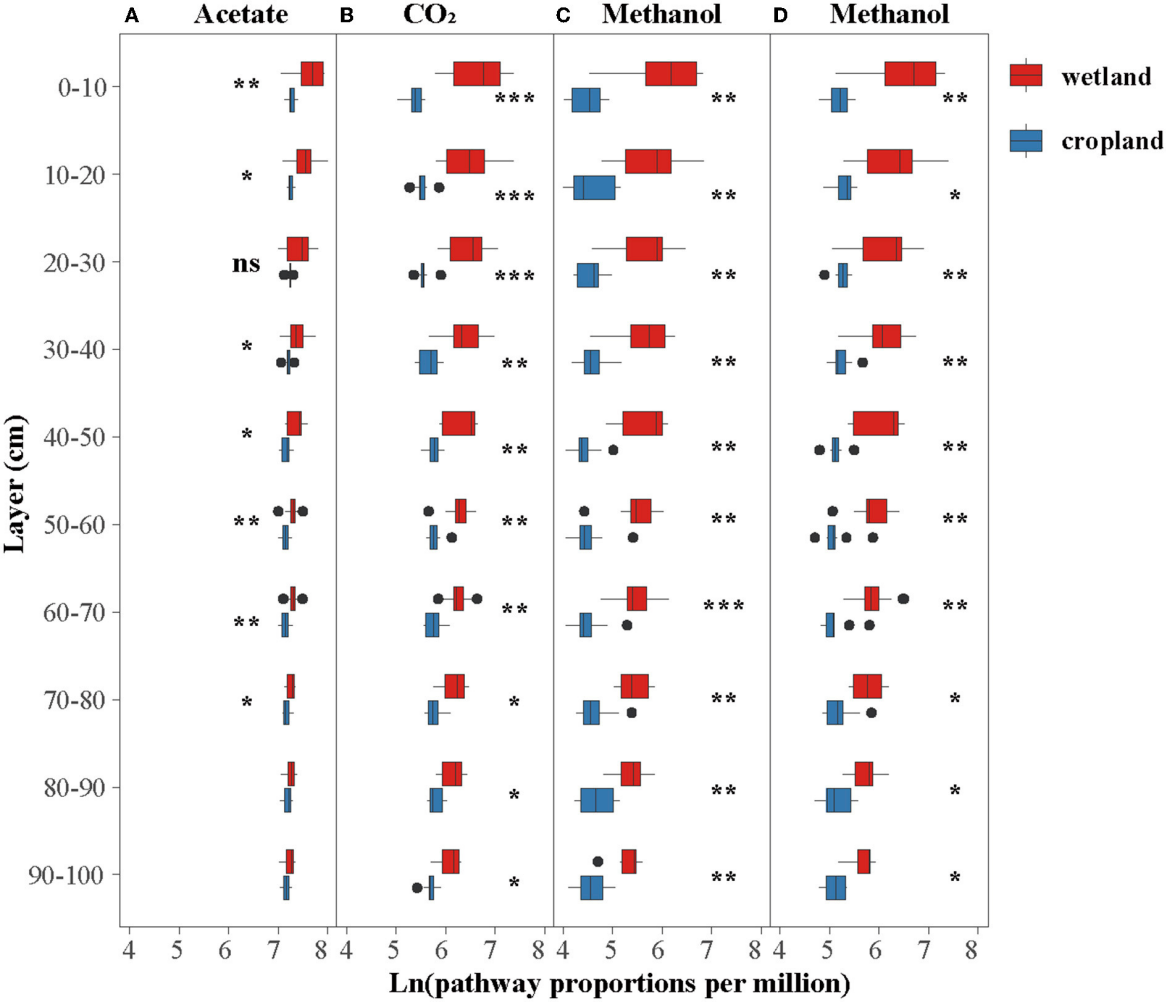
Cultivation effects on the proportion of genes in four methane production pathways and layers. **(A)** Acetate pathway, **(B)** CO_2_ pathway, **(C)** methanol pathway, and **(D)** methyl pathway. “*,” “**,” and “***” indicate a significantly difference between wetland and cropland (Kruskal–Wallis test, *p* < 0.05, *p* < 0.001, *p* < 0.0001, respectively).

The abundance of four methanogenic pathway genes were significantly decreased in cropland versus wetland (*p* < 0.05, Figure 1D and Supplementary Figure S1). Comparing with natural wetland, the functional module of the acetate (M00357), CO_2_ (M00567), methanol (M00356), and methylamines pathways (M00563) decreased by about 19, 52, 68, and 62% in cropland, respectively (Figure 1D). The response ratio of 12 functional genes involved in four pathways confirmed the suppression after wetland cultivation (Supplementary Figure S1B). Moreover, the suppression effects of cultivation on the abundances of CO_2_, methanol, and methylamines pathway genes were also significant across four seasons while with different extents (Figure 2). The lowest extent of these three pathways were 21, 31, and 23% in autumn and the highest of 62, 81, and 75% in spring, respectively. At the same time, the reductions of the acetate pathway were only significant in winter (27%), spring (27%), and summer (24%) (Figure 2). The abundances of CO_2_, methanol, and methylamines pathway genes also decreased significantly in all layers, with the extents along with the soil profile from 75, 83, and 79% at 0–10 cm to 41, 54, and 59% at 80–90 cm (Figure 3). The inhabitation of acetate pathway was significant from 0 to 10 to 60 to 70 cm, with the greatest at 0–10 cm (34%) and lightest at 50–60 cm (14%) (Figure 3).

**Figure 4:**
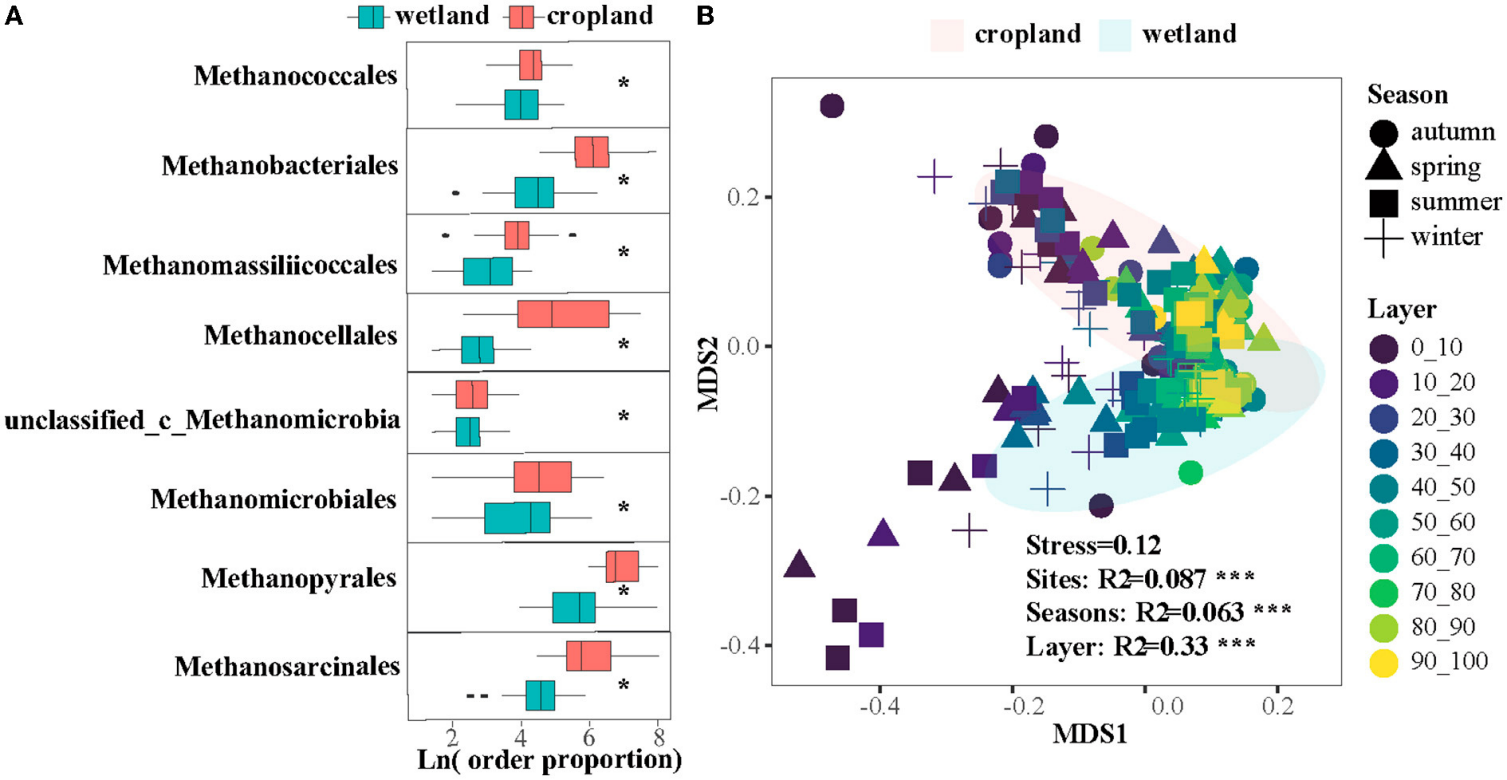
Cultivation effects on **(A)** 8 methanogenic orders and **(B)** community structure in order level using non-metric multidimensional scaling (NMDS). * indicate a significantly difference between wetland and cropland (Kruskal96Wallis test, *p* < 0.05).

The effects of cultivation on methanogenic composition and community structures were also significant (Figure 4). Eight orders of methanogens, including *Methanococcales, Methanobacteriales, Methanomassiliicoccales, Methanocellales, Methanomicrobiales, Methanopyrales, Methanosarcinales*, and one of unclassified order in *Methanomicrobia* class significantly declined after cultivation (Figure 4A, *p* < 0.05). Community structures of methanogens were significantly distinguished in wetland and cropland (Figure 4B, R^2^ = 0.087, *p* < 0.01), across seasons (Figure 4B, R^2^ = 0.063, *p* < 0.01) and along layers (Figure 4B, R^2^ = 0.33, *p* < 0.01).

We assumed that four pathways account for 100% of CH_4_ production, the percentages of four pathways were calculated and found a transition of CO_2_, methanol, and methylamines pathways to the acetate pathway after cultivation (**Figure 6**). The dominant path was acetate in the wetland, which accounted for 55% of total methanogenic gene abundance (**Figure 6A**). The CO_2_ pathway accounted for 20%, the methanol pathway accounted for 8%, and the methylamines pathway accounted 15%. In cropland, the percentage of acetate pathway increased to 70%, while CO_2_, methanol, and methylamines pathways decreased to 15, 6, and 9%, respectively (**Figure 6B**). Besides, the promotion of acetate percentage and the inhabitation of other three percentages in cultivated cropland were more remarkable in spring and summer than that in autumn and winter (Table 2 and Supplementary Figures S4, S6).

The cultivation influences on the percentages of four pathways were also different in wetland and cropland along soil profiles (Table 2 and Supplementary Figure S5). In the wetland, only the methylamines pathway percentage fluctuated along the soil profiles, while the percentages of acetate, CO_2_, and methanol pathways were kept vertically consistent (Table 2 and Supplementary Figure S5). In cropland, the percentage of the acetate pathway significantly decreased with depth, while the percentage of the CO_2_ pathway increased with depth (Table 2 and Supplementary Figures S5A,B). Methanol and methylamines pathways were consistent along the soil profiles in cropland (Table 2 and Supplementary Figures 5C,D).

### Four methanogenesis genes across seasons and along soil profiles

Seasonal fluctuation of four pathway genes was significantly different in wetland and cropland. In the wetland, the abundances of all four pathways were significantly different in four seasons (Table 1, *p* < 0.05), with the highest in spring and lowest in autumn (Supplementary Figure S2). However, the CO_2_ pathway varied insignificantly across seasons in cropland (Table 1, *p* = 0.60). Abundances of acetate, methanol, and methylamines pathways significantly changed across four seasons in cropland (Table 1, *p* < 0.05), with the highest in winter and lowest in autumn for acetate pathway, while highest in autumn and lowest in spring for methanol and methylamines pathways (Supplementary Figure S2).

**Table 1:** The Kruskal–Wallis test of functional genes in four pathways.

**Pathway**	**Wetland**				**Cropland**			
	**Season**		**Layer**		**Season**		**Layer**	
	**χ^2^**	***P***	**χ^2^**	***P***	**χ^2^**	***P***	**χ^2^**	***P***
Acetate	60.74	**<0.0001**	25.18	**0.0028**	8.11	**0.044**	32.13	**0.00019**
CO_2_	65.77	**<0.0001**	16.58	0.056	2.06	0.60	52.11	**<0.0001**
Methanol	62.36	**<0.0001**	15.92	0.069	47.28	**<0.0001**	5.11	0.82
Methyl	62.59	**<0.0001**	21.47	**0.011**	32.31	**<0.0001**	13.26	0.15

Significantly differences are highlighted by bold value.

The abundances of the four pathways also varied along with the soil profiles (Table 1 and Supplementary Figure S3). In the wetland, the abundance of acetate and methylamines pathways significantly decreased along with the soil profiles (Table 1 and Supplementary Figure S3, *p* < 0.05). At the same time, the fluctuations in the abundance of CO_2_ and methanol pathways along soil profiles were insignificant (Table 1, *p* = 0.056 and *p* = 0.069, respectively). In cropland, acetate and CO_2_ paths were remarkably different along with soil profiles (Table 1, *p* < 0.05), with the highest at 0–10 cm and 40–50 cm, while the lowest at 50–60 and 0–10 cm, respectively (Supplementary Figure S3). The methanol and methylamines pathway genes were steady along the soil profiles (Table 1).

### Correlations of functional genes, their proportions, with other factors, and CH_4_ production potential

**Figure 5:**
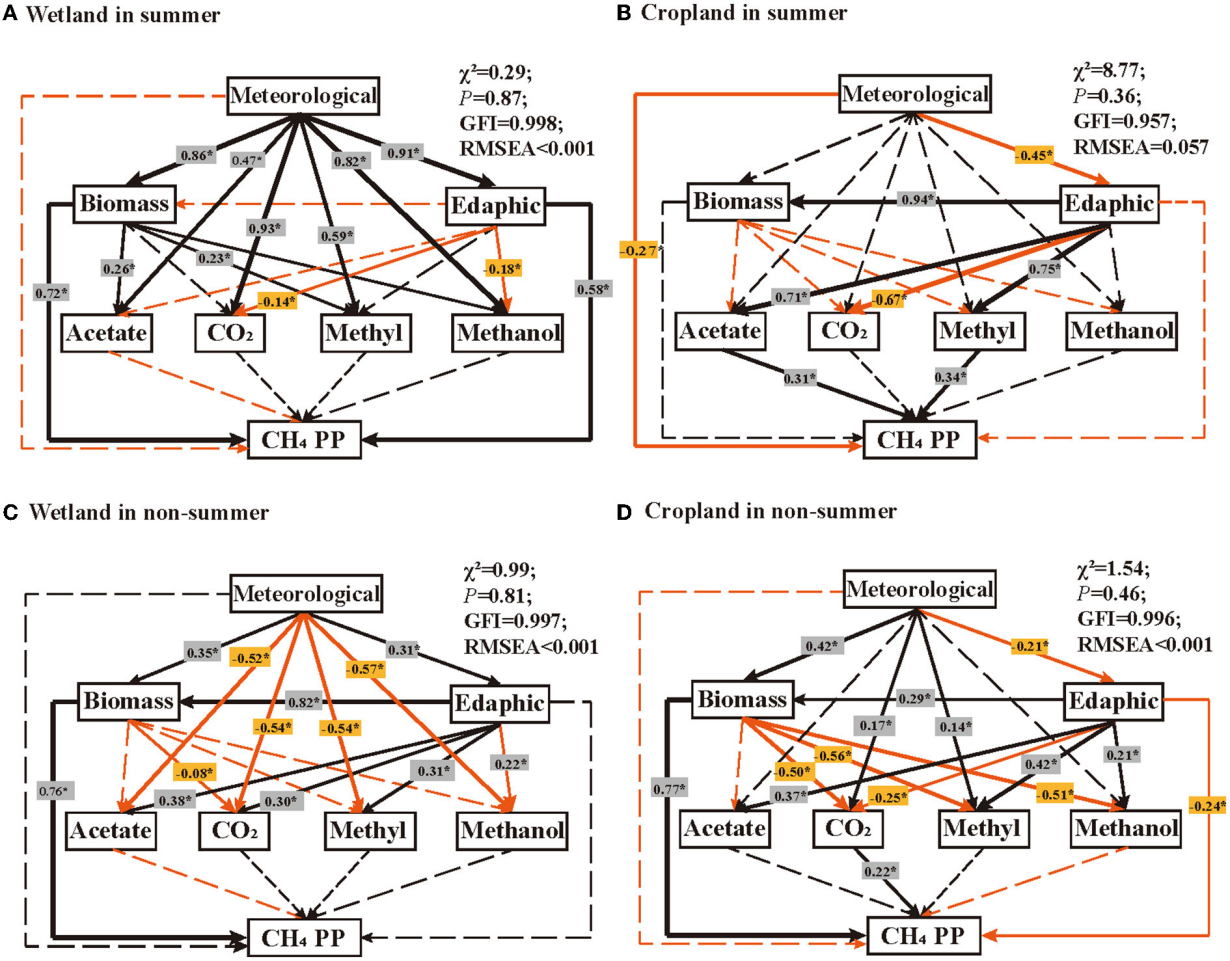
Structural equation modeling (SEM) illustrating the direct and indirect effects of physicochemical characteristics and functional genes on CH_4_ production potential (CH_4_ PP) in **(A)** wetland in summer; **(B)** cropland in summer, **(C)** wetland in non-summer, and **(D)** cropland in non-summer. Meteorologic characteristics refer to soil temperature and soil water content. Edaphic factors included T.C., T.N., T.P., T.S., and C/N ratio. Biomass is referred MBC, MBN, MBP, MBS, and MBC/MBN ratio. Arrows in black and red indicate positive and negative effects, respectively. Solid and dashed lines indicate significant and insignificant correlations, respectively. “*” indicates *p* < 0.05. R^2^ indicates that it appears near every response variable in the model. Model fitness details, including χ^2^, *p-value*, GFI, and RMSEA, are close to the figure.

SEMs showed that different characters correlated with CH_4_ production potential in wetland and cropland, and those relationships were also different in summer and non-summer (Figure 5). In the summer of wetland, soil microbial biomass and edaphic characters had strong positive correlations with CH_4_ production potential (Figure 5A, R^2^ = 0.72 and 0.58, respectively, *p* < 0.05). The micrometeorological character indirectly affected CH_4_ production potential through a strong positive effect on biomass and edaphic characters (R^2^ = 0.86 and 0.91, respectively, *p* < 0.05). Biomass, edaphic, and micrometeorological characters related to four pathways without altered CH_4_ production potential directly (Figure 5A). In the summer of cropland, micrometeorological character negatively correlated with CH_4_ production potential (Figure 5B, R^2^ = −0.27, *p* < 0.05), while acetate and methylamines pathways positively correlated with CH_4_ production potential (Figure 5B, R^2^ = 0.31 and 0.34, respectively, *p* < 0.05). In non-summer seasons, CH_4_ production potential was significantly related with biomass (R^2^ = 0.76, *p* < 0.05) and indirectly related with meteorological and edaphic characters in the wetland (Figure 5C). However, CH_4_ production potential in cropland were positively correlated biomass (R^2^ = 0.77, *p* < 0.05) and CO_2_ pathway genes (R^2^ = 0.22, *p* < 0.05), as well as negatively correlated with edaphic character (R^2^ = −0.24, *p* < 0.05) (Figure 5D).

**Figure 6:**
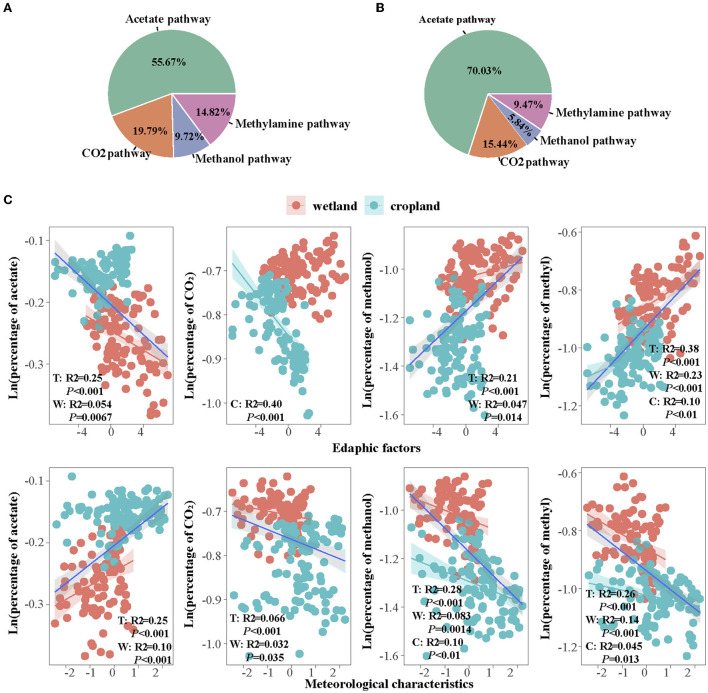
Percentage of four pathways in **(A)** wetland and **(B)** cropland; and the correlations of four pathway percentages with edaphic and meteorological factors **(C)**. T, total; W, wetland; C, cropland.

The regression of four pathway percentages with edaphic and meteorological characteristics showed that the relation of acetate and the other three pathway percentages were opposite (Figure 6C). The acetate percentage increased with meteorological characteristics overall and in the wetland (Figure 6C, R^2^ = 0.25 in total and R^2^ = 0.10 in wetland, *p* < 0.05), while decreased with edaphic characteristics (R^2^ = 0.25 in total and R^2^ = 0.054 in wetland, *p* < 0.05). However, the proportions of methanol and methylamines pathways increased with edaphic factors (R^2^ = 0.21 in total and R^2^ = 0.047 in wetland for methanol, R^2^ = 0.38 in total, R^2^ = 0.23 in wetland, and R^2^ = 0.10 in cropland for methylamines, *p* < 0.05) but decreased with meteorological characteristics (R^2^ = 0.28 in total, R^2^ = 0.083 in wetland, and R^2^ = 0.10 in cropland for methanol, R^2^ = 0.26 in total, R^2^ = 0.14 in wetland, and R^2^ = 0.045 in cropland for methylamines, *p* < 0.05). The proportion of CO_2_ pathway declined with both edaphic (only significant in cropland, R^2^ = 0.40 *p* < 0.05) and meteorological characteristics (R^2^ = 0.066 overall, and R^2^ = 0.032 in wetland, *p* < 0.05).

## Discussion

### Cultivation's suppressing effect on methanogenesis genes across seasons and depth

Four methanogenesis pathways (Figure 1D) and eight dominant orders of methanogens were suppressed in cultivated cropland (Figure 4A). The same pattern was found in both CH_4_ flux and production potential (Figures 1B,C), and confirmed by a global synthesis (Figure 1A). These suppressions were significant across seasons and soil profiles for genes in CO_2_, methanol, and methylamines pathways (Figures 2, 3). The reduction effect of cultivation on methanogens and methanogenic functional genes was also found in other field studies (Liu et al., 2018, 2019). It could be explained by the development of aerobic microorganisms in cultivated cropland (Pankhurst et al., 2002), while the flooded condition in the wetland was a benefit for methanogens (Stres et al., 2008; Watanabe et al., 2009). The different effects of micrometeorological characters on functional genes in the wetland (significant) and cropland (insignificant) (Figure 5) also indirectly confirmed the critical role of soil water condition and temperature in regulating methanogenesis in land-use change. Cultivation also decreased soil T.C., MBC, soil microbial abundance, and enzyme activities (Song et al., 2012; Zhu et al., 2021). Altogether, the coordinated inhibition of substrate, methanogenic genes, and methanogens activities, which might be controlled by meteorological characters, suppressed the potential of CH_4_ production in cropland.

The declining of gene abundances in four methanogenesis pathways after cultivation differed across seasons, with the largest in spring/summer and the lightest in autumn (Figure 2). It reflected different seasonal patterns of functional genes in wetland and cultivated cropland (Supplementary Figure S2), which had also been proved by other field studies (Breidenbach and Conrad, 2015; Ji et al., 2019; Gontijo et al., 2020). Moreover, the seasonal variations of the CO_2_ pathway and its percentage were significant in wetlands but not in cropland (Figure 2, Supplementary Figures S2, S4, and Tables 1, 2), which was incongruous with our second speculation. Furthermore, the more remarkable decline of soil water content in spring and summer than in other seasons was consistent with the fact that the inhibition of four pathway genes was more remarkable in those two seasons (Supplementary Figure S7). Previous studies observed that temperature and soil water conditions would impact soil microbes and CH_4_ emissions (Zhang et al., 2015; Chang et al., 2020; Chen et al., 2020). While other studies also proved that flooded conditions influence methanogenic communities (Breidenbach and Conrad, 2015). Our results also proved that both the four pathways and CH_4_ production potential were correlated with soil micrometeorological characteristics (Figures 5, 6). In reality, soil water condition and temperature were more like two interrelated characteristics. Thus, we would conclude that the different temperature fluctuations and soil water conditions regulated the different seasonal fluctuation patterns of methanogenic genes in wetland and cropland, which further determined the different response patterns of methanogenesis genes to cultivation.

**Table 2:** The Kruskal–Wallis test of four methane production pathways' percentage.

**Pathway**	**Wetland**				**Cropland**			
	**Season**		**Layer**		**Season**		**Layer**	
	**χ^2^**	***P***	**χ^2^**	***P***	**χ^2^**	***P***	**χ^2^**	***P***
Acetate	60.04	**<0.0001**	10.97	0.28	29.46	**<0.0001**	31.10	**0.00028**
CO_2_	50.41	**<0.0001**	6.23	0.72	1.54	0.67	64.38	**<0.0001**
Methanol	52.78	**<0.0001**	7.80	0.55	56.08	**<0.0001**	6.10	0.73
Methyl	56.84	**<0.0001**	21.42	**0.011**	40.09	**<0.0001**	10.17	0.34

Significantly differences are highlighted by bold value.

The inhibition effects of cultivation were also remarkably different along with soil profiles, with more extensive surface layers such as 0 to 30 cm than those in the subsurface layers (Figure 2). The influence of cultivation on methanogenic functional genes along soil profiles was also proved by our previous study on microbial biomass characteristics (Zhu et al., 2021) and several other research (He et al., 2017; Jerman et al., 2017). The cultivation mainly affected surface soil and generated less dissimilar soil microbial biomass, community structure, and functional genes. Compared with wetland and other natural ecosystems soil, suppression of cultivation on soil organic matter content, carbon stocks, labile carbon, and C/N ration were also more impressive in the surface layers than that in the subsurface layers. It should be noted that the reduction of methanogenic functional genes of CO_2_, methanol, and methylamines pathways were also significantly in deep layers from 60 to 100 cm (Figure 2). Moreover, the functional genes of the CO_2_ pathway increased in the middle layers from 30 to 100 cm compared with the surface layers from 0 to 30 cm in cropland (Figure 2). It suggests that cultivation could also redistribute functional genes within the soil profiles. Redistribution of active genes might result from soil carbon redistribution and influence soil carbon sequestration potential along the soil profiles (Wiesmeier et al., 2012).

### Varied land conversion impacts four methanogenesis pathways

We found that the dominant methanogenic pathway was the acetate pathway, followed by the CO_2_ pathway, methylamines pathway, and methanol pathway in both land-use types (Figure 4). The percentage of acetate pathway was 56% in wetland and 70% in cropland, which were consistent with quantification of methanogenic pathways using stable carbon isotopic methods, with acetate pathway contributing 50–80% to the CH_4_ production (Conrad, 1999, 2005; Metje and Frenzel, 2005; Zhang et al., 2015). The contribution of the CO_2_ pathway to CH_4_ production remains significant uncertainty. A previous study proved that 35–44% of the CH_4_ output was observed from the CO_2_ pathway in peatland (Kotsyurbenko et al., 2004). Hydrogenotrophic methanogenesis was also observed as the most dominant pathway in forest wetlands (Wu et al., 2021). In our study, the percentage of CO_2_ pathway genes accounted for 20% in wetland and 15% in cropland (Figures 4A,B). Different ecosystem and vegetation types might be the reason leading to the vast difference in the CO_2_ pathway.

A previous study using stable-isotope analyses proved that methanol and methylamines were the basis of <5 and 1% of the methane produced in the freshwater wetland (Lovley and Klug, 1983). Our results found a higher percentage of methylamines (15% in wetland and 9% in cropland) and methanol pathways (10% in wetland and 6% in cropland) (Figure 4). Narrowe et al. (2019) found that methylotrophic methanogenesis was present and active in freshwater wetland, accounting for about 8% of *mcrA* transcripts in Ohio, USA. Higher contributions of methylotrophic methanogenesis, especially using methanol as substrate, were also observed in the Zoige wetland on the Tibetan plateau (Jiang et al., 2010). It confirms the contributions of methylotrophic methanogenesis to methane production, which might be underestimated. It also indicates that it is necessary to consider more than acetoclastic and hydrogenotrophic methanogenesis in biogeochemical models of methanogenesis.

The percentage of acetate pathway genes increased while the other three pathways decreased after cultivation in our study (Figures 6A,B, Supplementary Figure S6). It indicates that the acetate pathway is the dominant pathway to produce CH_4_ in wetlands and would be enhanced after cultivation. It is also consistent with other studies on Ljubljana marsh (Jerman et al., 2009) and temperate climatic Marsh (Hornibrook et al., 2000). However, it challenges the suspect that CH_4_ from high latitude wetlands is not derived from acetate (Hines et al., 2001), and hydrogenotrophic methanogenesis accounts for 80% of the total methanogenesis and accompany acetate consumed by syntrophic oxidation to butyrate (Metje and Frenzel, 2005). Moreover, the changes in abundances of methanol and methylamines pathways were larger than acetate and CO_2_ and acetate pathways after conversion of wetland to cropland, which decreased by 40% for methanol pathway and 36% for methylamines pathway, while 22% for CO_2_ pathway and 26% for acetate pathway in cropland (Figures 6A,B). It suggests that the methanol and methylamines pathways might be important in regulating CH_4_ production during land-use change even though their percentages are lower than the other two pathways. It is worth further exploration (Vanwonterghem et al., 2016).

The unbalance transition of the acetate pathway and the other three pathways were also observed in other studies and explained by the temperature plant-induced substrate availability (Zhang et al., 2018; Chen et al., 2020). In our study, the percentage of the acetate pathway was positively correlated with micrometeorological character and negatively correlated with edaphic factors, while the other three pathways were opposite (Figure 6C). These results were consistent with our suspicion that acetoclastic methanogenesis was more sensitive to substrates, which are active in conditions with higher bioavailability (simple and label), but the percentage of hydrogenotrophic methanogenesis was more sensitive to temperature, which increased with temperature (Breidenbach and Conrad, 2015; Zhang et al., 2015; Conrad, 2020). The unbalance transition was also observed across seasons and along soil profiles. The enhancement of the acetate pathway and the inhabitation of the other three pathways were more remarkable in spring and summer, as well as in surface soil layers from 0 to 30 cm (Supplementary Figures S4, S5). As the CH_4_ productions were more impressive in spring and summer, as well as surface soil layers, it confirmed that the regulations of pathways of CO_2_, methanol, and methylamine pathways were outstanding even in their less abundance.

### Correlation between functional genes and CH_4_ production potential in cropland other than in wetland

We found that functional genes were dominant in regulating CH_4_ production potential in cropland while not significant in the wetland (Figure 5). The decoupling of functional genes from their real functions was also observed in methane-cycling in the wetland (Ho et al., 2013; Narrowe et al., 2019) and paddy-upland soil (Liu et al., 2018). Functional genes determined in this study are DNA-based, while their active components might be also restrained by the real-time environment (Liu et al., 2020). It reminds us that mRNA-based analysis such as metatranscriptomics needs to be developed and should be involved in future studies. The inconsistency of the relationship between the abundance of four pathway genes and CH_4_ production potential was also observed in the summer and non-summer seasons in cropland. The CH_4_ production potential was positively correlated with acetate and methylamines pathways in summer, while significantly correlated with the CO_2_ pathway in non-summer seasons (Figure 5). It confirmed the imbalance seasonal fluctuation patterns of four pathway genes so that their different relationships with CH4 production potential in summer and non-summer in cropland to some extent. It confirmed the imbalance seasonal fluctuation patterns of four pathway genes so that their relationships with CH_4_ production potential in cropland to some extent.

The factors that affect the potential of CH_4_ production were different in the wetland and cropland. Biomass and edaphic characters remarkably affect CH_4_ production potential in the wetland in summer and non-summer seasons (Figures 5A,C). The positive correlations between soil microbial biomass/soil carbon and CH_4_ production are widely observed (Altor and Mitsch, 2008; Weedon et al., 2013; Zhang et al., 2019). As the primary indicators of soil quality and health, soil microbial biomass and edaphic characters would represent substrate quality and quantity (Xu et al., 2013). Thus, we can suspect that substrate would be the limiting factor to CH_4_ production in wetlands, proving the high CH_4_ cost of sequestering carbon in the wetland (Hemes et al., 2018). However, the aerobic of cropland soil would alleviate the substrate limitation because the temporary exposure of soil to oxygen could stimulate carbon degradation (Wilmoth et al., 2021). This biogeochemical compromise between high CH_4_ emission in wetland and high carbon loss in cropland would also be impressive and worth quantitative estimating in the cultivation and restoration process.

## Conclusion

Our results provided genomic evidence of the suppression impact of land conversion on four methanogenic pathways. Specifically, four pathway genes were inhibited by land-use modification, with different fluctuation patterns across seasons and along soil profiles in wetland and cropland. In wetlands, the abundance of four pathway genes was the highest in spring and the lowest in autumn, while cropland was highest in winter and lowest in spring. The abundance of four pathways also declined along the soil profiles in wetland but not in cropland. Those patterns react as a more remarkable inhibition of functional genes in spring/summer and surface soil layers such as 0 to 30 cm. The acetate pathway was dominant in both wetland and cropland, while changes in the abundance of the other three pathways and their ratios were larger than the acetate pathway after cultivation. Furthermore, the relative contributions of the acetate pathway were also enhanced by soil micrometeorological characters in cultivated cropland, synchronized with the limitation of contributions of the other three pathways, which were suppressed by soil micrometeorology. These findings implicate the importance of distinguishing the CH_4_ production pathways across seasons and along the soil profiles and their responses to environmental change (Xu et al., 2016).

This study represents one of the first attempts to distinguish four dominant methanogenic pathways when evaluating land conversion impacts along soil profiles at a seasonal scale. The quantitative information of the methanogenic pathways is critical for benchmarking the ecosystem models for more accurately simulating the CH_4_ mechanisms in response to environmental change. It raises the feasibility of integrating four methanogenesis pathways into CH_4_ cycling modeling.

## Data availability statement

The datasets presented in this study can be found in online repositories. The names of the repository/repositories and accession number(s) can be found at: NCBI—PRJNA853804.

## Author contributions

CS and XX conceived the project. XZ, YZ, and CG carried out soil sampling. XZ carried out lab experiments. NW carried out data analysis and interpretation with all other authors. NW and XX wrote the manuscript with assistance from other co-authors. All authors contributed to the article and approved the submitted version.

## Funding

This study was partially supported by the Strategic Priority Research Program of the Chinese Academy of Sciences (Grant No. XDA28020502), the Top Notch program in China, the National Key R&D Program (2016YFA0602303), the National Natural Science Foundation (Nos. 41730643, 41701198, and 41401106) of China, Ecology Innovation Team (2020CXTD02) in Minzu University of China, and Northeast Institute of Geography and Agroecology, Chinese Academy of Sciences. This study was partially supported by the National Natural Science Foundation of China (No. 32171873).

## Conflict of interest

The authors declare that the research was conducted in the absence of any commercial or financial relationships that could be construed as a potential conflict of interest.

## Publisher's note

All claims expressed in this article are solely those of the authors and do not necessarily represent those of their affiliated organizations, or those of the publisher, the editors and the reviewers. Any product that may be evaluated in this article, or claim that may be made by its manufacturer, is not guaranteed or endorsed by the publisher.
