# Supplementary material for: Metagenomic evidence of suppressed methanogenic pathways along soil profile after wetland conversion to cropland
Source: Front Microbiol. 2022 Sep 20;13:930694. doi: 10.3389/fmicb.2022.930694 (PMC9530824; doi:10.3389/fmicb.2022.930694)
Supplement: Supplementary file 1 [file Data_Sheet_1.docx]

**Genomic evidence of suppressed methanogenic pathways along soil profile after wetland conversion to cropland**

Nannan Wang^1*^, Xinhao Zhu^1^, Yunjiang Zuo^1,2^, Jianzhao Liu^1,2^, Fenghui Yuan^3^, Ziyu Guo^1^, Lihua Zhang^4^, Ying Sun^1^, Chao Gong^1^, Changchun Song^1*^, Xiaofeng Xu^5*^

1 Key Laboratory of Wetland Ecology and Environment, Northeast Institute of Geography and Agroecology, Chinese Academy of Sciences, Changchun 130102, Jilin, China

2 University of Chinese Academy of Sciences, Beijing, 100080, China

3 Department of Soil, Water, and Climate, University of Minnesota, St. Paul, 55108, USA

4 College of Life and Environmental Sciences (Minzu University of China), Beijing 100081, China

5 Biology Department, San Diego State University, San Diego, CA 92182, USA

Corresponding authors: N.W., [wangnannan@iga.ac.cn](mailto:wangnannan@iga.ac.cn); X.X. [xxu@sdsu.edu](mailto:xxu@sdsu.edu)

**Figure S1**. Four methanogenic pathway genes across seasons. Different letters indicate significantly different (Dunn’s-test, *P* < 0.05) in different seasons.

**
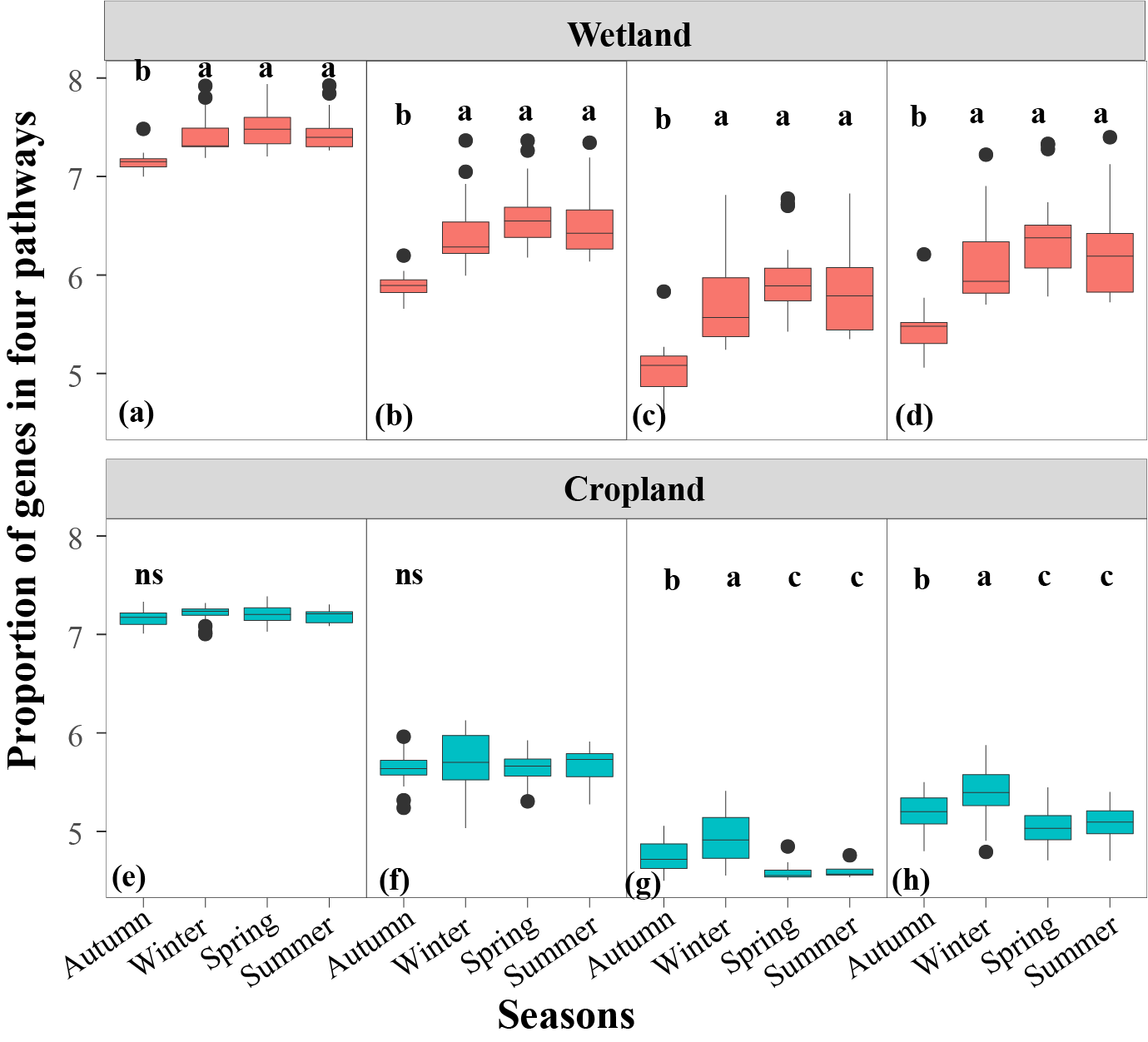
**

**Figure S2**. Four methanogenic pathway genes along soil profile. Different letters indicate significantly different (Dunn’s-test, *P* < 0.05) in soil layers.


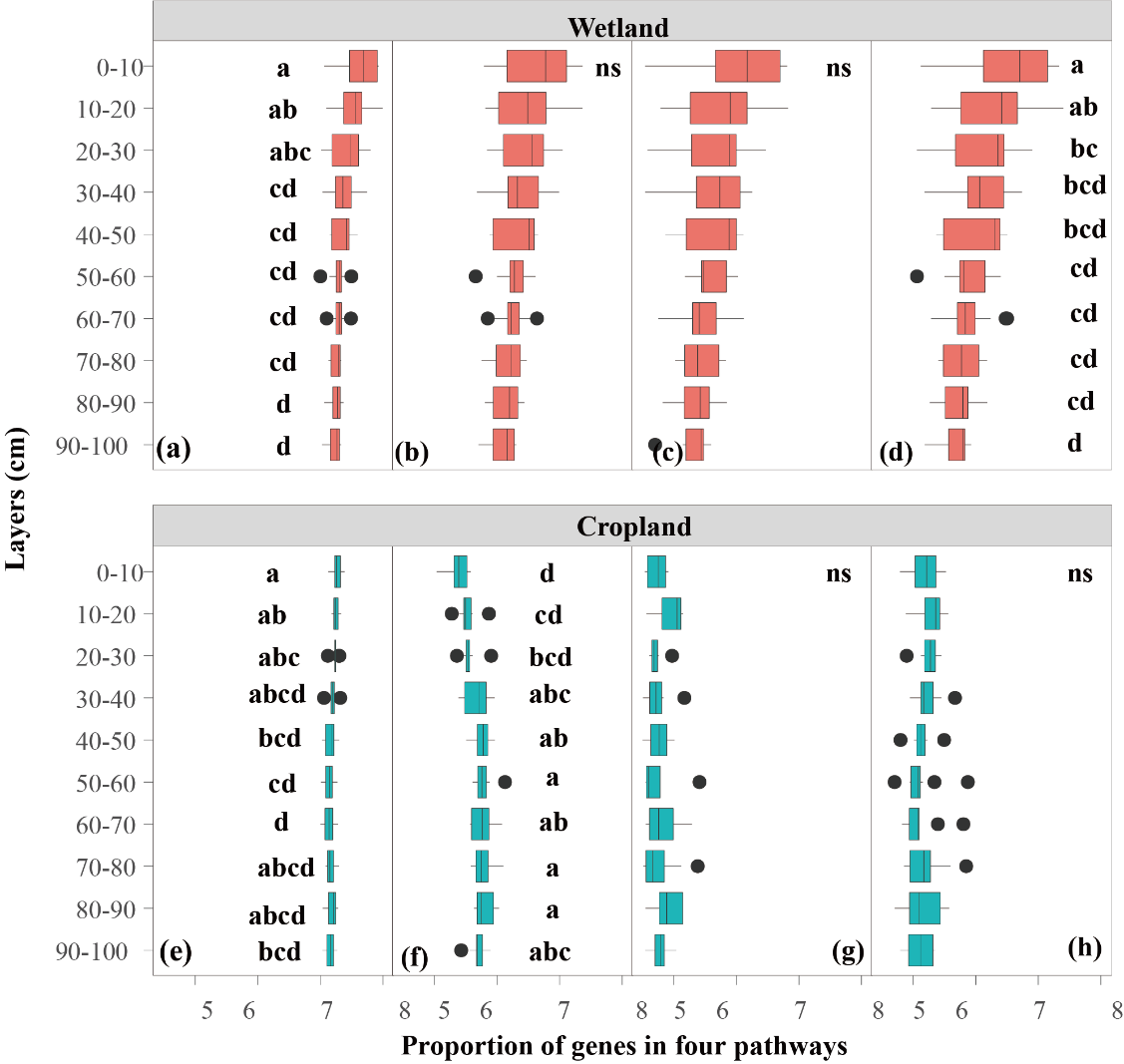


**Figure S3.** Seasonal changes of percentage of four methanogenic pathway genes. “*” indicates significantly different between wetland and cropland (Kruskal-Wallis test, *P* < 0.05).


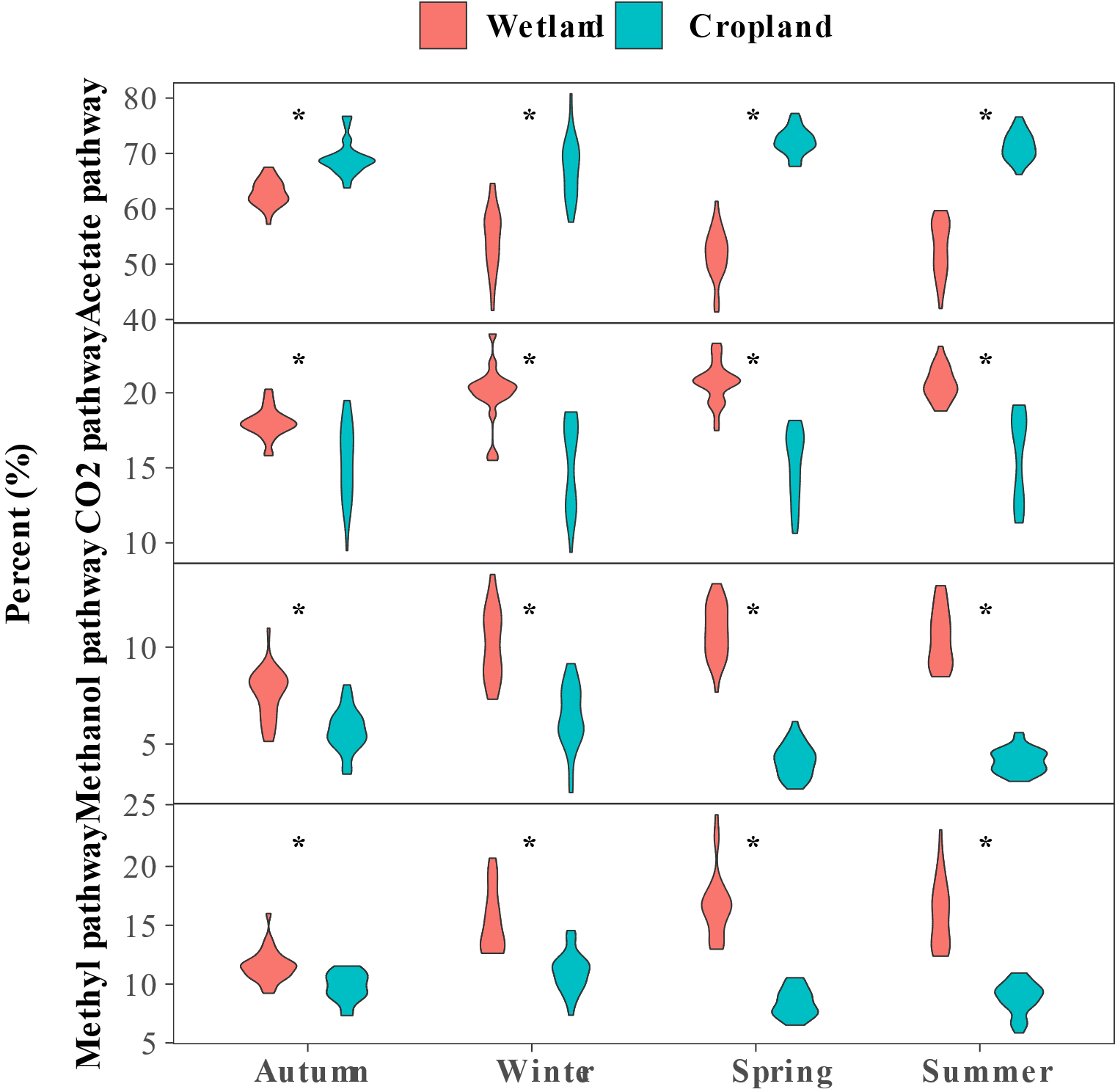


**Figure S4**. Percentages of four methanogenic pathway genes along soil profile. “*” indicates significantly different between wetland and cropland (Kruskal-Wallis test, *P* < 0.05).


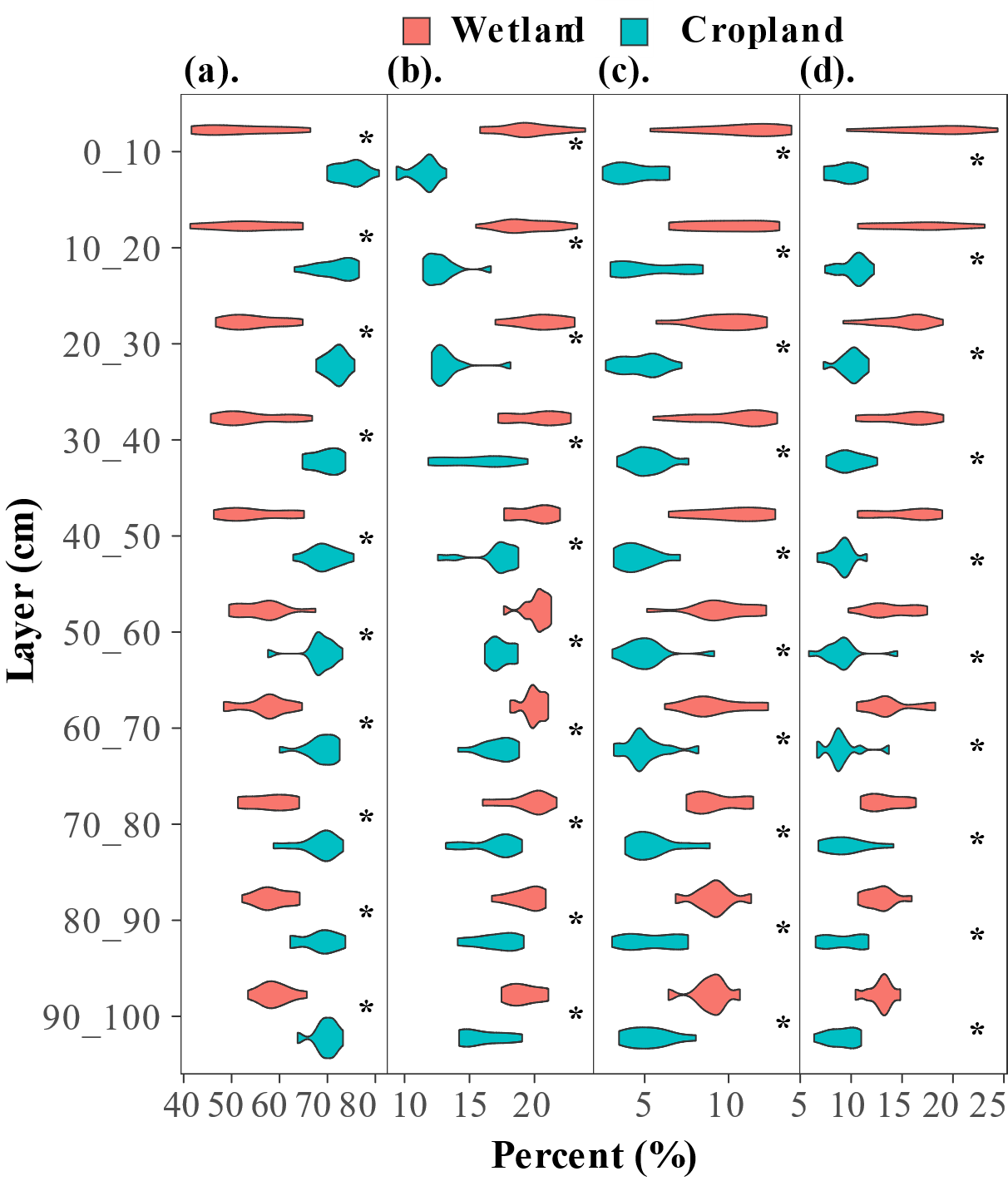


**Figure S5** Percentages of four pathways across four seasons in wetland and cropland.


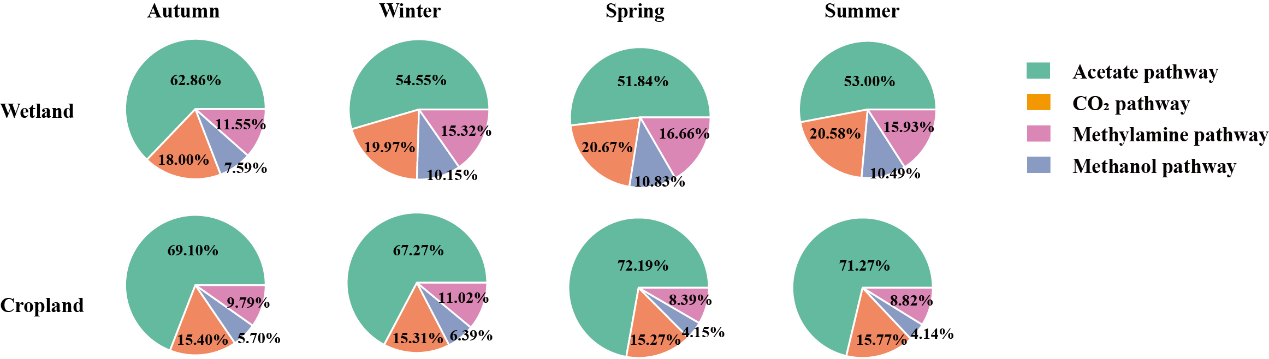


**Figure S5** Soil water content in wetland and cropland along soil profile in four seasons.


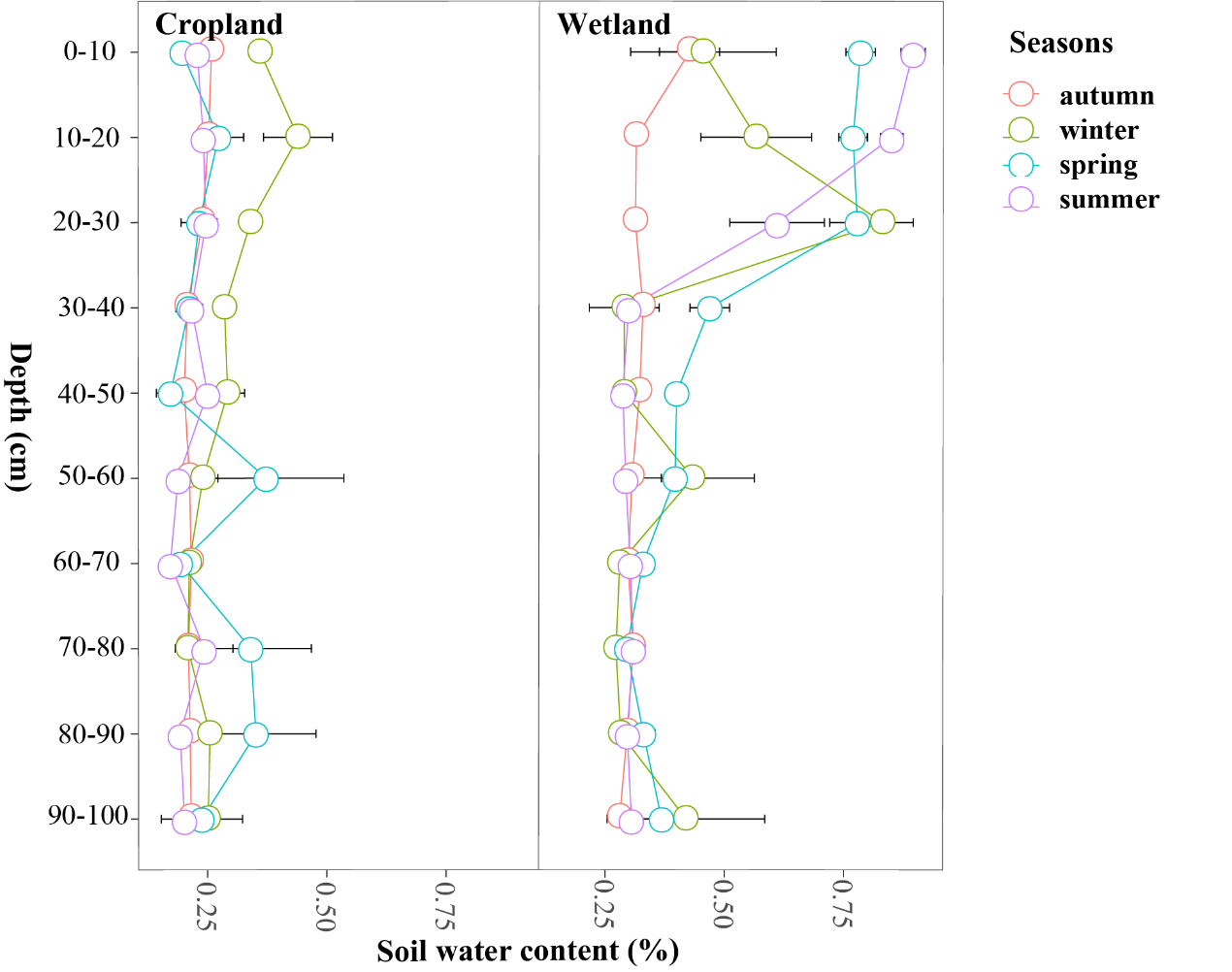


**Table S1.** The Kruskal-Wallis test of functional genes in four pathways across seasons.

| **Pathway** | **Seasons** | **Wetland vs Cropland** | |
| --- | --- | --- | --- |
|  |  | χ^2^ | *p* |
| **Acetate pathway** | Autumn | 1.36 | 0.24 |
|  | Winter | 19.39 | **<0.0001** |
|  | Spring | 35.68 | **<0.0001** |
|  | Summer | 39.67 | **<0.0001** |
| **CO_2_ pathway** | Autumn | 26.47 | **<0.0001** |
|  | Winter | 29.42 | **<0.0001** |
|  | Spring | 44.26 | **<0.0001** |
|  | Summer | 44.26 | **<0.0001** |
| **Methanol pathway** | Autumn | 20.07 | **<0.0001** |
|  | Winter | 28.42 | **<0.0001** |
|  | Spring | 44.26 | **<0.0001** |
|  | Summer | 44.26 | **<0.0001** |
| **Methyl pathway** | Autumn | 17.88 | **<0.0001** |
|  | Winter | 26.95 | **<0.0001** |
|  | Spring | 44.26 | **<0.0001** |
|  | Summer | 44.26 | **<0.0001** |

**Table S2**. The Kruskal-Wallis test of functional genes in four pathways across depth.

| **layers** | **Acetate pathway** | | **CO_2_ pathway** | | **Methanol pathway** | | **Methyl pathway** | |
| --- | --- | --- | --- | --- | --- | --- | --- | --- |
|  | χ^2^ | *p* | χ^2^ | *p* | χ^2^ | *p* | χ^2^ | *p* |
| **0_10** | 9.01 | **0.0027** | 17.28 | **<0.0001** | 14.96 | **0.00011** | 14.08 | **0.00017** |
| **10_20** | 5.60 | **0.018** | 16.33 | **<0.0001** | 13.65 | **0.00022** | 10.83 | **0.0010** |
| **20_30** | 2.43 | 0.12 | 16.33 | **<0.0001** | 14.08 | **0.00017** | 12.40 | **0.00043** |
| **30_40** | 5.33 | **0.021** | 13.23 | **0.00028** | 13.65 | **0.00022** | 14.08 | **0.00017** |
| **40_50** | 4.85 | **0.028** | 11.64 | **0.00065** | 14.19 | **0.00017** | 13.14 | **0.00029** |
| **50_60** | 7.69 | **0.0055** | 12.02 | **0.00053** | 13.25 | **0.00027** | 11.62 | **0.00065** |
| **60_70** | 9.38 | **0.0022** | 14.55 | **0.00014** | 15.44 | **<0.0001** | 14.99 | **0.00011** |
| **70_80** | 5.84 | **0.0163** | 10.23 | **0.0014** | 11.16 | **0.00084** | 10.23 | **0.0014** |
| **80_90** | 3.42 | 0.064 | 9.36 | **0.0022** | 12.16 | **0.00049** | 9.36 | **0.0022** |
| **90_100** | 2.12 | 0.15 | 10.39 | **0.0013** | 10.97 | **0.00093** | 10.39 | **0.0013** |
